# Future Perfect? The Future of the Social Sciences in Public Health

**DOI:** 10.3389/fpubh.2017.00357

**Published:** 2018-01-10

**Authors:** Rachel C. Shelton, Mark L. Hatzenbuehler, Ronald Bayer, Lisa R. Metsch

**Affiliations:** ^1^Department of Sociomedical Sciences, Columbia University Mailman School of Public Health, New York City, NY, United States

**Keywords:** public health, training, public health practice, social sciences, interdisciplinary

## Abstract

This is a critical and perhaps unprecedented time for the social sciences in public health. While there are many opportunities for the social sciences to continue making transformative contributions to improve population health, there are significant challenges in doing so, particularly in a rapidly changing political landscape. Such challenges are both external (e.g., congressional calls for reducing social science funding) and internal (e.g., scholars criticizing the social sciences for being stagnant and siloed). This paper highlights four key tensions that the field is grappling with and that have direct implications for how to train the next generation of social scientists in public health. We also discuss how departmental and institutional decisions made in response to these tensions will determine how the social sciences in public health are ultimately recognized, sustained, and advanced.

The social sciences^1^ have made profound contributions to population health. While the specific theories and methods that each discipline employs vary, the value of the social sciences relates in part to their common focus on identifying and addressing persistent social realities and inequalities, and shared interest in advancing understanding of social forces that shape population health. The contributions of social science research to population health include (but are certainly not limited to): the health consequences of stigma, prejudice, and discrimination (1, 2); the impact of socioeconomic position (3), stress (4), social networks (5), social support (6), and place (7) in shaping health and health inequalities; the role of policy, power, and politics in structuring the health of populations (8–10); and the consideration of social context in the development and implementation of multilevel interventions that improve population health (11–13).

While there are many opportunities for the social sciences to continue making transformative contributions to improve population health, there are also significant challenges to sustaining and expanding these contributions. Challenges come from both within and outside the academy. External challenges include recent congressional calls for significantly reducing, even eliminating, funding for the social sciences, potentially due to perceptions that the social sciences are too “soft” and too “liberal” (14). These threats are fueled, in part, by a growing anti-science, anti-expertise discourse in certain segments of the American population. There has been heightened uncertainty among social scientists about how the social sciences will be valued and funded in light of recent political changes, making this a critical time to examine its value and contribution in public health (15).

Challenges also come from within the social sciences. In a widely discussed OpEd in *The New York Times*, Yale physician and sociologist Nicholas Christakis excoriated the social sciences, claiming that the lack of change in social science departments and disciplines is “counterproductive, constraining engagement with the scientific cutting edge and stifling the creation of new and useful knowledge” (16). Failure to alter the “basic DNA of the social sciences” might, according to Christakis, “result in having the natural sciences co-opt topics rightly and beneficially in the purview of the social sciences” (16). Other examples of the de-valuing of the social sciences include the large investments being made in biomedicine (e.g., precision medicine, clinical healthcare) over population health (17).

To successfully navigate these challenges, we argue that the field must confront several key tensions that we and others have identified. In the sections that follow, we highlight four such tensions, including (1) the balance of disciplinary versus interdisciplinary structures; (2) the contribution of the social sciences in framing the questions asked to advance public health; (3) the translation of research beyond academia to impact population health; and (4) the funding and institutional sustainability of the social sciences in public health.^2^ While this is not an exhaustive list, it represents tensions that the field is currently grappling with and that have direct implications for how to train the next generation of social scientists in public health. We also raise additional issues with which the field must grapple beyond training, particularly in the context of a rapidly changing political landscape that may de-value the social sciences. We approach the topics raised below from our perspective as scholars representing diverse social science disciplines within public health (including psychology, sociology, political science, and behavioral science).

Our goal in this paper is not to resolve the tensions that we highlight (which may, in some instances, not be feasible or desirable). Nor is our goal to take a side in the debates over these tensions. Not only do we view such debates as healthy and necessary to advance the field but we also see the varying perspectives we present as having value and import within the field. In fact, the diversity of perspectives has, in many cases, contributed to the success of the social sciences in advancing population health research. Our more modest aim, instead, is to explicitly highlight how departmental and institutional decisions that are made in response to these tensions will, by necessity, shape how we train the next generation of social scientists in public health. In turn, these decisions will help to determine how the social sciences in public health are ultimately recognized, sustained, and advanced.

## Consideration of Discipline Versus Interdisciplinary Structures and Foundations

The first key tension relates to what drives interdisciplinary versus disciplinary work within public health, and the appropriate role of traditional social science disciplines within institutions that conduct interdisciplinary research and training in public health. Currently, there are few structures in place to facilitate and maintain traditional social science disciplines in public health, and there is diversity in how this is addressed institutionally. For example, some social science programs that focus on population health are within arts and sciences departments (e.g., The Center for Medicine, Health, and Society at Vanderbilt University), whereas other social science programs that focus on public health are housed within schools of public health (e.g., the Department of Sociomedical Sciences at Columbia’s Mailman School of Public Health) (18).

While there appear to be multiple models for integrating the social sciences in public health within academic institutions, many departments and schools have placed less investment in hiring various discipline-specific social scientists (e.g., historians, anthropologists); in contrast, they have relatively more representation from behavioral scientists, some of whom may be trained more generally in social and behavioral science and do not have discipline-specific social science degrees. Furthermore, in our experience, blurred boundaries sometimes exist between the type of training someone receives, and the department in which they are based (e.g., a sociologist could be based in a department of social/behavioral sciences or in a department of epidemiology with a strong social epidemiology focus).

This tension has important implications for how best to train the next generation of social scientists within public health. There are currently two main approaches. One takes the perspective of advancing scholarship within social science disciplines. According to this line of thinking, it is critical that social scientists are first trained and mentored in their respective disciplinary departments and that disciplinary connections are maintained. Social science departments provide a foundation in the theories and methods that define their disciplines. This disciplinary grounding may allow social scientists to provide insights into how to address important public health issues, while concurrently contributing to the advancement of their respective disciplines.

Despite strengths of this approach, there are potential drawbacks. For instance, traditional social science departments may not perceive public health research as addressing the key questions these disciplines are pursuing, or as advancing discipline-specific theory or methods. In some cases, maintaining connections with the discipline may impede the ability to address issues that are most relevant for public health. For example, the social sciences outside of public health may focus on more technical disciplinary-specific concerns, which may deflect attention away from tackling the issues most relevant to public health. Addressing this issue may require a return to the roots of the social sciences that focused on addressing persistent fundamental social problems such as poverty and inequality.

The second approach is for public health researchers to obtain training through an interdisciplinary degree rather than through a social science department. According to adherents of this model, conducting rigorous interdisciplinary work that includes theories, methods, and approaches from across the social sciences is paramount to effectively addressing public health issues and stimulating innovation. There are various programs that train the next generation in this interdisciplinary science approach [for a review of programs, see Ref. (18)]. For example, the Robert Wood Johnson Foundation developed the Health and Society Scholars training program, the first postdoctoral program explicitly devoted to population health. Scholars came from a range of social science disciplines, and through weekly seminars led by interdisciplinary faculty, received training in expanding cross-disciplinary thinking, developing collaborative competencies, and acquiring shared language across disciplines (18, 19). Although no longer in existence, one of the program’s most important legacies was changing the institutional culture and structure of the sites where the program was based; disciplinary silos were slowly broken down, new interdisciplinary research teams were created, and the value of interdisciplinarity was institutionalized (18, 19).

At the same time, numerous challenges can impede this kind of training. For example, interdisciplinary public health research often takes place in institutions in which the social sciences are not necessarily nourished. Different languages, values, and methods can also impede interdisciplinary work; furthermore, structural conditions within disciplines and institutions (e.g., expectations for tenure/promotion; funding structures; administrative or physical space barriers), and constraints on time to foster such relationships, can make this collaboration difficult. Additional questions for the social sciences in public health to consider related to these disciplinary/interdisciplinary issues are provided in Table 1.

**Table 1:** Critical questions for the social sciences in public health: implications for training the next generation.

*Questions pertaining to structural and disciplinary factors shaping social science research in public health*
What drives social scientists’ interests in public health, and how does this differ from what drives disciplinary work? For instance, public health asks, “How can anthropology, sociology, history, economics, or political science help to answer the problem of obesity or AIDS?” In contrast, in disciplinary work in social sciences, researchers ask, “How can the study of obesity or AIDS help advance sociology, anthropology, or political science?” What are the intersections between the social sciences in public health and the fields of social epidemiology and behavioral science? How can schools best incentivize and embrace interdisciplinarity? What are the tensions between disciplinary needs of the social sciences and the research needs of public health and how should that dynamic be negotiated? Is a school of public health the right space to be addressing some of these challenges? What new structures are needed (e.g., training, new disciplines) to ensure that social scientists stay relevant in an increasingly biomedical reality? How might different social science disciplines tackle some of these issues in public health?

*Questions pertaining to the content and contribution of the social sciences in public health*
Does the desire to do policy-relevant research, which means doing research that policy-makers will find useful, limit the kinds of questions asked? What counts as “evidence” in policy, practice, and among the social sciences? And how does that shape the kind of research that social scientists in public health conduct? When social scientists enter the world of policy-making, do they feel constrained to be candid about the extent to which there are uncertainties about the evidence that is driving public policy? What is the relationship between trying to advance a policy agenda and the recognition of uncertainty? Should social scientists seek out research projects that are likely to have tangible policy implications (i.e., the concept of “strategic science”)? How does such research differ from more theoretical and less policy-relevant studies that often characterize social science inequities? When, why, and how is academic policy research most likely to have a policy impact?

*Questions pertaining to the dissemination and communication of social science research to the public and policy-makers*
What is the role of academics and social scientists in ensuring that evidence-based programs or policies are disseminated and implemented? How much and what kind of evidence is needed for an intervention or policy to be disseminated or more broadly implemented and scaled up? What is the boundary between research dissemination, knowledge translation, and advocacy in the work of social scientists in public health and what is their responsibility in this domain? What is the role of social scientists in public health in ensuring that their research is translated for use by policymakers and the public? Beyond policy-making, what are the other critical ways social scientists can make an impact on population health? How can social scientists leverage their role as communication and dissemination experts among biomedical-focused researchers and scientists to disseminate biomedical innovations?

*Questions pertaining to funding the social sciences in public health*
Are there certain research questions that should be asked but cannot be asked because of the current funding environment? In the current soft-money environment in most schools of public health, what is the future of the social sciences in universities concerned with public health as we move forward? What models of teaching, education, and research will sustain the social sciences in public health in this environment? What is our role as social scientists in confronting a world of social inequality, particularly in light of how funding and grants are structured (e.g., short-term, disease-specific)? How does the structure of funding at NIH and CDC as organized by diseases impact the funding of social science research and advancements in science? Are there situations in which it is appropriate for social scientists to take funding from corporations to support their research programs in public health?

## Content and Scope of Social Science Research in Public Health

A second key tension pertains to the role of the social sciences in framing the questions asked to advance public health, and whether social science research should focus on issues perceived to have high policy relevance. The “strategic science” approach systematically directs scholarship to have more purposeful and timely policy impact in an effort to facilitate evidence-based policy-making (20). Strategic science has been defined as “research designed to address gaps in knowledge important to policy decisions, derived from the reciprocal flow of information between researchers and policy makers, and communicated not only in scholarly publications but also in forms relevant to policy makers” [(20), p. 2445]. In one example of this approach, Roberto et al. (21) tested the effectiveness of adding the total number of recommended calories (2,000 cal) in menu labels, a topic being considered by policy-makers. The researchers took their findings to policy-makers, who included the findings as part of the national menu-labeling policy and the Affordable Care Act.

An alternative approach to strategic science is to pursue research that may not address immediate policy questions but that nonetheless advances scholarship, theory, and the knowledge base on a particular topic or discipline. This approach is exemplified by Meyer’s (22) work on minority stress theory. When he started this work in the 1990s, the stated goal was knowledge production—i.e., to develop a theory that described the role of social stressors in shaping sexual orientation health disparities. It was not clear when, or whether, this work would be used to shape policy. However, this research ultimately had tremendous policy relevance in 2008, when it was cited in the U.S. Court of Appeals’ decision that rendered Proposition 8 (a California ballot proposition that would have eliminated the right of same-sex couples to marry) unconstitutional.

These two approaches have significant implications for how training is approached. Doctoral- and MPH-level practitioners often lack the skills to translate their research into evidence-based policy, given that formal training in public health typically contains insufficient emphasis on policy-related competencies. Moving forward, a key decision will be whether to provide a foundation in policy-relevant training, including writing policy briefs, policy translation, communicating research evidence to policy-makers, and creating multi-sectorial teams. One potential solution could include a cross-university seminar for doctoral students to train them on policy translation in an apprenticeship model. Additional questions related to this topic are provided in Table 1.

## Dissemination and Communication of Social Science Research in Public Health

Another related tension concerns the role of social scientists in translating and disseminating research outside of academia (23). Dissemination has been defined as “an active approach of spreading evidence-based information to the target audience *via* determined channels using planned strategies” [(24), p. 118]. The scientific community lacks consensus on the appropriate role of researchers in knowledge translation, or appropriate boundaries between research, policy-making, and advocacy (18, 25). Some argue that social scientists should not engage in the translation and dissemination of the work, which is viewed by some as inappropriately veering on advocacy. There have historically been some concerns in academia that disseminating work to policy-makers, key stakeholders, and the broader public impinges upon perceived scientific neutrality; moreover, social scientists are often not trained to communicate beyond academia, and some posit that it is not their responsibility to do so (25).

A contrasting viewpoint is that a key responsibility of social scientists is to communicate new innovations within larger networks, contexts, and systems in a way that values social science (26). To address this responsibility, a science of dissemination and implementation has been developing in recent years (27). Brownson et al. (28), for instance, have identified a range of systems and structural strategies to encourage research dissemination, including providing academic incentives (e.g., related to tenure) and requiring a dissemination plan in funding announcements. A continuum of how researchers can engage in varying degrees to translate their research has been proposed, ranging from raising awareness of an issue (e.g., publishing scientific articles, presenting at meetings, writing a press release or popular piece) to communicating findings to policymakers (e.g., developing short policy summaries, providing testimony at hearings) to actively lobbying on behalf of particular issue (e.g., media advocacy, writing letters to the editor, meeting with elected officials) (29).

Moving forward, it will be critical to more formally decide how to address this tension, particularly as the field considers the kinds of training provided to MPH and doctoral students and whether to develop competencies to enhance dissemination and communication of research (e.g., media training, writing for popular press, social media dissemination, communicating with stakeholders, engaging with knowledge brokers) (18). Additional competencies to facilitate interdisciplinary collaboration and team science—important skills for conducting interdisciplinary research and disseminating findings to key stakeholders outside of academia—could include training in maintaining group dynamics, conflict management, and stakeholder communication (18). Training in knowledge translation has implications not only for enhancing scientific communication with the media, policy-makers, and the public but also for improving knowledge translation and communication between researchers (18). An example is The OpEd Project’s Public Voices Fellowship that matches researchers with high-level journalist mentors to write opinion pieces for major media platforms including NPR, the *Washington Post*, and *Time* (30). However, there has not been a widespread integration of this area into the curricula at schools of public health, despite its growing presence at NIH (31, 32). Table 1 provides additional questions to consider regarding dissemination of research and knowledge translation.

## Institutional Sustainability and Funding of the Social Sciences in Public Health

A final tension, particularly felt in the current funding and political context, relates to the future role and contribution of the social sciences in “soft-money” environments (see Table 1). Because public health schools are soft-money institutions, they are dependent on external grant support. For the social sciences, this poses a serious challenge. Especially vulnerable are history and anthropology, which tend to receive fewer NIH grants than other disciplines. Private foundations that may support anthropology, history, and political science do not carry the institutional overhead support crucial to institutional sustainability. In those social sciences with access to NIH support, the current funding environment is still perilous. Because of the current prospects for funding, many public health schools have begun to consider the option of corporate support for research projects. While this may contribute to the resolution of budgetary difficulties, it inevitably raises critical ethical issues about corporations shaping research agendas, the viability of research undertakings that do not obtain corporate support, and the obvious problems of reputational risk, even when conflicts of interest are considered (33). This tension has ramifications for the type of grant-writing taught in formal coursework, training, and mentoring.

## Future Directions for Social Sciences in Public Health

In this article, we have discussed critical challenges that are relevant to training the next generation of social scientists in public health. These include tensions related to interdisciplinarity and disciplinary structures and foundations, how social scientists prioritize and value research focused on advancing social science theory and knowledge versus research with high policy relevance, the direction social scientists take with regard to dissemination and translation of research for broader consumption, and how to achieve institutional sustainability. The diversity of perspectives that we have represented across these tensions has contributed to the richness of the social sciences in public health. As one example, strategic science that explicitly seeks to answer policy-relevant questions, as well as knowledge production that seeks to address gaps in scientific understanding, both add value to the field of public health. Consequently, we are not advocating for one approach over another. Instead, our goal is to highlight these tensions and diverse perspectives so that we can more explicitly recognize them and the implications they have for a range of departmental and institutional decisions.

The tensions we have highlighted here have critical implications for the structure and training provided at public health schools, the direction of social science research that the next generation of social scientists in public health will pursue, the dissemination and reach of their research, and our ability to support and fund them. As noted at the outset, we highlight issues related to training because whether and how these tensions are confronted will have direct implications for decisions made about training the next generation of social scientists in public health. Issues of training are particularly timely given that the Council on Education for Public Health (CEPH) Accreditation for Schools of Public Health has recently updated its criteria to focus more on skill-building (versus topical areas) in its coursework (34). As such, our discussion of training future scholars may suggest additional ways of addressing curricular changes required by CEPH.

The title of this paper is a play on the future perfect tense, the idea that an action will have been completed (finished or “perfected”) at some point in the future. We aspire toward a more perfect future—one in which the contributions of the social sciences in public health can be more fully realized to improve population health and reduce health inequalities. There are no easy solutions to guarantee this perfect future. But to sustain and advance their rich history and contributions, it is critical that leaders in social sciences within public health confront the challenging issues and questions raised in this article and their implications for training the next generation.

## Author Contributions

RS and MH took the lead in writing sections of the paper. LM and RB contributed to the writing of section of the paper and provided comments on all sections and drafts of the paper. All authors reviewed the final version of the paper and approve the content of the work.

## Conflict of Interest Statement

The authors declare that the research was conducted in the absence of any commercial or financial relationships that could be construed as a potential conflict of interest.

## Footnotes

^1^For the purposes of the paper, we define social sciences broadly to include multiple disciplines, including, but not limited to, history, political science, anthropology, sociology, economics, and psychology. While we acknowledge that there are differences among these disciplines, for the purposes of the paper we are less interested in those distinctions than in conceptualizing the social sciences in contrast to other approaches (e.g., biomedical approaches).

^2^The tensions we have raised typically represent the polarities of these debates (e.g., interdisciplinarity versus disciplinary). While we acknowledge that these extremes may not capture the modal practice, they serve an illustrative purpose because they highlight some of the key assumptions and tensions underlying these debates.
